# Editorial: Mind-body medicine and its impacts on psychological networks, quality of life, and health

**DOI:** 10.3389/fnint.2023.1188638

**Published:** 2023-04-04

**Authors:** Steffen Schulz, Dirk Cysarz, Georg Seifert

**Affiliations:** ^1^Department of Pediatrics, Division of Oncology and Hematology, Charité – Universitätsmedizin Berlin, Freie Universität Berlin, Humboldt-Universität zu Berlin, Berlin Institute of Health, Berlin, Germany; ^2^Integrated Curriculum for Anthroposophic Medicine, Faculty of Health, Witten/Herdecke University, Witten, Germany; ^3^Department of Pediatrics, Faculty of Medicine, University of São Paulo, São Paulo, Brazil

**Keywords:** mind-body medicine, health, emotions, lifestyle and behavior, networks, psychology

Living conditions in industrialized countries have led to a significant increase in life expectancy in recent decades. Likewise, the proportion of chronic diseases is growing. This includes cardiovascular diseases, chronic pain, inflammatory bowel diseases, and cancer. Unfavorable lifestyle factors, such as accumulative stress, lack of exercise, and poor nutrition, compounded by the persistent imbalance between exertion and recovery, lead to the manifestation and chronification of disease. A fundamental awareness of the connection between our mind, emotions, lifestyle, and health has grown. Understanding of body and mind interaction is increasing and is supported and confirmed by evidence, demonstrating a high level of clinical relevance. It is out of this field of research that mind–body medicine (MBM) has developed. MBM targets the interplay of body, mind, emotions, and behavior, extending to the regulation of vegetative physiological signaling pathways. The goal of MBM is to use scientifically-based knowledge on the interactions between body, mind, and emotions to promote salutogenetically-based resources. In this context, the body, mind, and emotions are regarded as working in unity while illnesses can be understood as producing a disbalance in complex biopsychological systems. A central theme of MBM is the need for lifestyle changes. Psychological aspects of MBM affect lifestyle factors and thus indirectly affect many different areas of the mind-body connection. MBM techniques include mindfulness-based stress reduction, meditation, active movement, relaxation, mindfulness, stress management and relaxation, habits of perception, and evaluation among others. These work through complex biopsychological and social systems on different areas of lifestyle, providing a set of tools to increase the sense of self-efficacy and promoting health. The therapeutic approaches of MBM encompass a variety of different methods that often facilitate cost-effective preventive or therapeutic options. Relevant evidence-based data are available, and this is an area with high scientific and clinical growth potential for lifestyle change and prevention of some of the most important health issues. Complementary treatments, developed from the emotional, mental, social, spiritual and behavioral factors, are combined with a variety of conventional medicine practices to influence health. The analysis of these signaling pathways have structural, dynamic, and regulatory mechanisms, and the information transfer in healthy and diseased states, provides insights into the physiological structures and functions of the whole integrated system with its different types of interactions.

This Research Topic focuses on the evidence-based investigations based on physical exercise, integrative strategies for self-care, applications from traditional Chinese medicine, hypnotherapy, Ayurveda, relaxation, meditation methods, yoga practice, Qigong, Tai Chi, biofeedback interventions, implementation of digital health tools, evaluation of behavioral change techniques, mindful stress relief, cognitive restructuring, autogenic training, and health-impacting social support. This Research Topic has collected a diverse range of research content from multidisciplinary contributions in the field of MBM, ranging from comprehensive data analysis to clinical practice applications. This Research Topic included six original research articles, two clinical trials, one article of hypothesis and theory, and five reviews.

Jeitler et al. investigates the use of self-care and lifestyle interventions as well as mental/emotional state experienced during the COVID-19 pandemic via an online survey for 1,138 participants. Individual health promotion strategies, such as spending time in nature, increasing physical activity, using naturopathic remedies, consuming a plant-based diet, or practicing mind-body interventions feature predominantly with participants who are female, middle-aged, and well-educated. Most participants were found to demonstrate an overall balanced mental/emotional state (Jeitler et al.). Xie F. et al. evaluated the effect of a Qigong exercise, prolong life with nine turn method (PLWNT), with 90 participants experiencing chronic fatigue syndrome (CFS) as a complex illness of unknown etiology and mechanisms focusing on fatigue, sleep quality, depression, and anxiety symptoms. The PLWNT Qigong showed the potential to be an effective rehabilitation method for CFS symptoms, including fatigue, sleep disturbance, anxiety, and depression. Testing whether increases in heart rate variability (HRV) could be detected after a 6-week smartphone-based HRV-biofeedback training, Schumann et al. investigate whether reductions in rumination levels in depressed patients were accompanied by the change in HRV. They found a significant correlation between resting HRV and rumination levels and suggested that HRV biofeedback intervention can be applied to improve cardiovagal function and to reduce depressive symptoms, including self-rated rumination tendencies. Liang et al. explore the relationship between physical fitness, calcium intake, calorie intake, and adolescent mental health to promote a healthy lifestyle and preventing mental problems. They showed that adequate calcium intake and the improvement of cardiopulmonary fitness in adolescents aged 12–13 are essential for the good development of their mental health. An aspect of how asthma affects adults' quality of life through social, emotional, physical, and occupational impacts was highlighted by Kharaba et al. Furthermore, with a focus on the influence of family support on the healing of gastric ulcers caused by endoscopic submucosal dissection, Gao et al. found that the occurrence and degree of negative emotions such as psychological anxiety and depression, in addition to the occurrence of gastric pain, may be reduced.

In a pilot trial, a 60-h MBM and comprehensive lifestyle modification training program, Bauer et al. demonstrates findings over a 10-week-period for patients with Crohn's disease (CD) in rural regions under pandemic conditions, including practices for stress reduction, stress management, relaxation, mindfulness meditation, breathing, yoga, and Qigong, elements of cognitive behavioral therapy, psychoeducational approaches. Xie Y. J. et al. studied the efficacy and feasibility of 12 weeks of Tai Chi training as a prophylactic treatment to prevent migraine in Chinese women and showed a significant reduction in the frequency of migraine attacks.

In the hypothesis and theory paper by McParlin et al., an entwined model that combines touch for alignment and active inference is presented to explain how the brain develops “priors” necessary for the health care provider to engage with the patient effectively. Touch is surmised to play a crucial role in achieving successful clinical outcomes and adapting previous priors to create intertwined beliefs.

The systematic review by Mühlenpfordt et al. intends to summarize the typical indications and outcomes and to systematically assess the effectiveness and safety of external, touch-based applications of anthroposophic medicine (e.g., rhythmical massages, embrocations, and compresses) given as complementary treatment for various conditions. In both a systematic review and meta-analysis, the current state of research assesses the effectiveness of mindfulness-based interventions on the academic performance of students as measured by their grade point average (Ostermann et al.) while illuminating the remaining lack of clarity of the exact mechanisms of action. Another systematic review and meta-analysis by Wang et al. analyzes the risk of dementia or Parkinson's disease (PD) with individuals with Sjogren's syndrome (SS), finding in their analysis that people with SS are likely at higher risk of PD and dementia than the general population (Wang et al.). In a critical mini review (Leça and Tavares), scientific evidence for the use of mindfulness-based interventions (MBI) for fibromyalgia were evaluated and conclude that despite the sparsity of well-structured longitudinal studies, there are some promising results showing that the MBI are effective in reducing the negative aspects of fibromyalgia. In a narrative review (Esch and Stefano) the basic principles of MBM, including the introduction of a rational framework for the implementation of MBM-based interventions are presented through the BERN framework (behavior, exercise, relaxation, and nutrition). The BERN model aims to strengthen health and resilience, and reduce stress. The mechanisms of action of these processes involve the central nervous system reward systems and correlate with the placebo and self-healing pathways.

The objective of this Research Topic aspires to understand how different physiological control systems, from the mind to the body, interact and to improve quality of life and health. We aimed to provide a foundation of evidence for new theoretical and practical approaches to solving problems and challenges in MBM. Contributing to a comprehensive overall picture of MBM, this unique set of multidisciplinary approaches and visions will expand the knowledge of integrative medicine and have a positive impact on future therapeutic developments.

## Author contributions

SS, GS, and DC: conceptualization, funding acquisition, roles/writing—editorial, and writing—review and editing. All authors contributed to the article and approved the submitted version.

